# Detection of genotype-1 of dengue virus serotype 3 for the first time and complete genome analysis of dengue viruses during the 2018 epidemic in Mandalay, Upper Myanmar

**DOI:** 10.1371/journal.pone.0251314

**Published:** 2021-06-04

**Authors:** Mya Myat Ngwe Tun, Aung Kyaw Kyaw, Takeshi Nabeshima, Aung Min Soe, Khine Mya Nwe, Kyaw Ko Ko Htet, Thet Htoo Aung, Thein Thein Htwe, Thidar Aung, Su Su Myaing, Tu Tu Mar, Ei Phyu Lwin, Hlaing Myat Thu, Kyaw Zin Thant, Kouichi Morita

**Affiliations:** 1 Department of Virology, Institute of Tropical Medicine, Nagasaki University, Nagasaki, Japan; 2 Department of Medical Research, Ministry of Health and Sports, Yangon, Myanmar; 3 Department of Medical Services, 550 Bedded Children Hospital (Mandalay), Ministry of Health and Sports, Mandalay, Myanmar; 4 Myanmar Academy of Medical Science, Yangon, Myanmar; Shanghai Public Health Clinical Center, CHINA

## Abstract

**Background:**

Dengue (DEN) is a neglected tropical disease, and surveillance of dengue virus (DENV) serotypes and genotypes is critical for the early detection of outbreaks. Risk factors for outbreaks include the emergence of new genotypes and serotype shifting.

**Methodology and principal findings:**

To understand the genomic and viral characteristics of DENV-infected patients, we conducted a cross-sectional descriptive study among pediatric patients admitted at the 550-bedded Mandalay Children Hospital during the 2018 DEN endemic season. We conducted virus isolation, serological tests, viremia level measurement, and whole-genome sequencing. Among the 202 serum samples, we detected 85 samples with DENV (46 DENV-1, 10 DENV-3, 26 DENV-4 and three multiple serotype co-infections) via reverse transcription quantitative/real-time PCR (RT-qPCR), and we obtained 49 DENV isolates (31 DENV-1, 10 DENV-3 and 8 DEN-4). We did not detect DENV-2 in this study. The viral genome levels in serum did not differ significantly among virus serotypes, infection status (primary versus secondary) and disease severity. Based on the phylogenetic analysis, we identified DENV-1 genotype-1, DENV-4 genotype-1 and DENV-3 genotype-3 and genotype-1 which was detected for the first time. Next-generation sequencing analysis revealed greater frequencies of nonsynonymous and synonymous mutations per gene in the nonstructural genes. Moreover, mutation rates were also higher among DENV-1.

**Conclusion/Significance:**

In conclusion, there was an increasing trend of DENV-3 cases during DENV endemic season in 2018 with the first detection of the genotype 1. However, DENV-1 has remained the predominant serotype in this study area since 2013, and we identified stop codon mutations in the DENV-1 genome. This report is the first to feature a complete genome analysis of the strains of DENV-3 and DENV-4 circulating among pediatric patients in Myanmar. This study highlighted the importance of annual surveillance for a better understanding of the molecular epidemiology of DENVs.

## Introduction

Dengue (DEN) is a neglected tropical disease that still constitutes a major public health problem in low-resource countries. *Aedes* mosquitoes (*Aedes aegypti* and *Aedes albopictus*) provide the main vectors for transmission of the diseases and 215 countries are suitable for survival and transmission of arboviral diseases [1]. The DEN virus (DENV) belongs to genus *Flavivirus*, a group of positive-sense, single-stranded RNA viruses, and it has four distinct yet closely related serotypes.

DENV infection is endemic in more than 128 countries, including Myanmar. The incidence of DEN cases is increasing, and the virus has spread to new areas worldwide. According to estimates from one mathematical model of DENV infection, about 390 million people (95% confidence interval; 284–528 million) contract DEN [2]. During the past two decades the number of reported cases has increased eightfold worldwide. According to the WHO report, the total number of DEN cases increased from 505,430 cases in 2000 to 4.2 million in 2019. Seventy percent of infections occur mainly in Asia [3].

In Myanmar. DEN results in high mortality among children. The Vector Borne Disease Control Program, under Myanmar’s Ministry of Health and Sports, routinely performs community-based vector control activities and the National Health Laboratory conducts surveillance of DENV serotypes and monitors the outbreaks. In Myanmar, the largest DENV outbreak occurred in 2015, when a record of 42,913 infections were reported across the country. However, the case fatality rate has steadily decreased and remains less than one percent [4].

Monitoring and surveillance of DENV serotypes are important for DENV prevention and control. In 2013 and 2015, when a DENV outbreak occurred in Mandalay, DENV-1 was predominant, and serotypes DENV-2 and DENV-4 also caused many infections. However, no DENV-3 was detected [4, 5]. One study conducted in 2006, detected DENV-3 in Mandalay, but this serotype remained undetected in Mandalay for more than a decade afterward [6]. Natural changes in DENV serotypes and genotypes constitute risk factors that engender outbreaks. DENV is still the public health problem and one of high mortality diseases among children and DENV serotype and genotype surveillance among children has been continuously performed since 2006. Here, we conducted hospital-based surveillance at the 550-bedded Mandalay Children Hospital during the 2018 DEN peak seasons to explore the virological characteristics of DENV serotypes among children in Mandalay and conduct a complete genome analysis of DENVs isolated in this study.

## Materials and methods

### Ethics statement

This study was a collaborative study between the Department of Medical Research and the Institute of Tropical Medicine, Nagasaki University under an agreement between the two institutes. Therefore, the ethical approvals were received from Ethics Review Committee of Department of Medical Research, Myanmar (083/2018) and Ethics Review Committee of Institute of Tropical Medicine, Nagasaki University, Japan (191003223). This study was conducted following the local regulation and laws after getting permission from the Department of Medical Research, Ministry of Health and Sports. This manuscript is also a joint publication with ethical authorship requirements between the Department of Virology, Nagasaki University and Department of Medical Research. We took written informed consent and assent from the parents and legally authorized representatives for taking blood samples from the participants.

### Sample size

Sample size calculation was done by using R software. We assumed that the proportion of virus isolation would be 40% based on similar previous studies conducted at the same study area in 2013 and 2015 and precision was taken as 0.07%. The confidence limit was 95% and calculated using the formula, N = Z^2^P (1-P)/ d^2^. Minimum sample size required for this study was 188 clinically diagnosed DEN patients and a total of 202 patients were recruited. All clinically diagnosed DEN patients (less than 13 years old) who were diagnosed by pediatricians and admitted to medical wards of study hospital were recruited. Critically ill patients under the care of Intensive Care Unit and patients who did not give consent and assent from legally authorized representatives were not included in this study.

### Patient recruitment

A cross-sectional descriptive study was conducted at the 550-bedded Mandalay Children Hospital, the tertiary center of pediatric care in Upper Myanmar where a total of 202 pediatric patients (less than 13 years old) who presented with fever and rash were recruited. This study was conducted at the peak seasons of DENV infection (July-August) in 2018. After getting consent from the legally authorized representatives, patients were examined by clinicians and their serum samples were collected and aliquoted for immediate screening and for storage at -80°C until use for other experiments. Screening of DENV infection (NS-1Ag, IgM and IgG) was performed in a point-of-care setting using CareUs Dengue Combo rapid test kit (WellsBio, Seoul, Korea) to detect DENV NS-1Ag, and IgM/IgG antibodies against DENV. This kit was validated for use in bedside settings in a previous study [7]. Following WHO recommendation, confirmation of DEN infection was done by a combination of different detection techniques such as virus isolation (gold standard), molecular method (RT-qPCR) and serological tests (NS-1 Ag, Anti-DENV IgM Ab). The WHO’s 2009 classification of DEN severity namely dengue without warning signs (DwoWS), dengue with warning signs (DwWS) and severe dengue (SD) was adopted [3].

### Serological tests

Serological tests were conducted to confirm DENV infection with the in-house DENV IgM capture ELISA and the in-house DENV IgG indirect ELISA. The in-house DENV IgM capture ELISA was performed following the procedures used in previous studies [8, 9]. Optical density (OD) was read at 492 nm and P/N (OD of positive control or sample/OD of negative control) ratio greater than and equal to 2 was considered as positive. To classify primary and secondary DENV infections, the in-house DENV IgG ELISA was performed. This in-house assay was validated in a previous study and showed very good correlation with the WHO’s standardized hemagglutination inhibition test was performed [10]. If the IgG titer was more than or equal to 29,000, infection was determined to be secondary, and if the titer was less than 29,000, the infection was considered primary.

### Virus isolation

Virus isolation was done at the Department of Virology, Nagasaki University, Japan. A serum sample (10 μL) was inoculated onto C6/36 *Aedes albopictus* mosquito E2 clone cells grown in flat culture tubes, and the inoculated cells were incubated at 28°C for 7 days for the first passage. Infected culture fluids (ICF) were harvested and kept at -80°C before the experiment. The second passage of the viral culture was performed using a fresh monolayer of confluent mosquito cell lines following the same procedure used in the first passage of virus culture [11].

### Conventional one-step RT-PCR

Viral RNA was extracted from ICF using the Qiagen Viral RNA extraction kit (Qiagen, Hilden, Germany). The presence of DENV genome was assessed by the conventional one-step RT-PCR, by using the Primescript TM One-step RT-PCR kit (Takara Bio Inc, Shiga, Japan), and with the DEN consensus primer set and methods as described in previous studies [5]. The DENV was serotyped using serotype specific primers in line with the methods in previous studies [5, 12, 13].

### Viral genome detection and measurement of DENV genome levels

Viral RNA was extracted directly from the serum sample using the same extraction kit used for ICF samples, a total 140 μl of serum was used to extract RNA. A reverse transcription quantitative/real-time PCR (RT-qPCR) was performed using serotype specific primers and probes validated in previous studies [14]. Amplification of the envelope protein gene was performed using TaqMan Fast Virus 1-Step Master Mix (Life Technologies, Carlsbad, CA) following the protocol from a previous report. Serial dilutions of standard cDNA (108–102 genome copies) were done and applied to quantify viral genome levels. The lowest detection limit for the viral genome was 100 copies. The viremia levels were described as log_10_ genome copies/mL.

### Whole genome sequencing and phylogenetic analysis

Whole-genome sequencing of isolated DENVs was performed by next-generation sequencing, Ion Proton (Life Technologies, CA, USA). Firstly, viral RNA was extracted from ICF using a Qiagen Viral RNA extraction Kit and whole transcriptome libraries (Ion Total RNA-Seq Kit v2, Life Technologies, CA, USA) were prepared. The low-quality reads (< 75% with quality score of < 20) were removed using FASTX-Toolkit Version (v) 0.0.14. A sequence quality check was done by FastQC v 0.11.8 before and after trimming the sequence. Trinity v 2.8.4 [15] for de novo assembly, Seqkit v 10.0.1 for sequence name repair, and blastn v 2.7.1 [16] was used for assembling de novo contig were used. Trimmed fastq data set were mapped by bwa v 0.7.17 [17] to the reference sequence chosen by blastn, and variants were detected by LoFreq v 2.1 3.1 [18], and Varscan v 2.4.3 [19]. From the output of Varscan, Samtools v 1.9 constructed the consensus dengue virus sequence. Data preprocessing was conducted according to the best practice workflow for GATK v 3.8.1 and Picard v 2.20. Using the International Nucleotide Sequence Database Collaboration data, DENV sequences were collected and annotated with Entrez-edirect and Seqkit. Alignment of the sequences of the coding region of whole DENV genome was done by Mafft v 7.407 [20]. Maximum-likelihood phylogenetic trees were constructed with Phyml v 3.2.0 [21]. Boot-strap values were obtained after 1,000 replications. The substitution model was selected by jModelTest v 2.1.10 [22]. All sequences were submitted to NCBI, GenBank with accession no (MW369303-MW369341, MW369428-MW369435).

### Statistical analysis

Data analysis was done with R software. For categorical variables, absolute number and percentage were described with a 95% confidence interval. A *p*-value less than 0.05% was used as the cutoff point for statistical tests significant. Student’s t-tests to compare two continuous variables: one-way analysis of variance for multiple-group comparisons and a chi-squared test for categorical variables were used.

## Results

### Study population, clinical presentation and laboratory test results

Of the 202 patients. 148 cases were confirmed as DENV infection based on serology and molecular methods as mentioned below. Following WHO’s 2009 classification, there were 22 DwoWS, 92 DwWS and 34 SD among the laboratory confirmed cases. The male-female ratio of these 148 confirmed DEN patients was 62:86 and the median age of the patients was 6.0 year (IQR 3.75–8.0). Of 202 patients with serum samples screened by CareUs DENV Combo test kits, 109/202 (53.9%) children were positive for NS-1 Ag, 90/202 (44.6%) cases for Anti-DENV IgM and 103/202 (50.9%) for anti-DENV IgG. A total 148 patients were confirmed to have DEN based on these laboratory tests. If the presence only of NS-1 Ag was used for diagnostic confirmation only 109 out of 148 (73.6%) case-patients could be confirmed. If the presence only of the virus genome or only of anti-DENV IgM were considered, only 85 case-patients (57.4%) or 90 case-patients (60.8%) could be confirmed. According to immune status of the patients, 56 cases were primary infection, and 85 cases were secondary infection. Seven confirmed DENV cases were not identified as primary or secondary infection because of the insufficient serum samples. Among the 34 cases of SD, nine patients (20.9%) had primary infection and 25 had secondary infection.

### Serotyping, isolation and virus quantification

By using RT-qPCR, 49 patients were positive for DENV-1, 11 patients for DENV-3 and 28 patients for DENV-4. There was a total of 85 patients that were RT-qPCR positives and three of them had different serotype co-infections (two patients infected with DENV-1 and DENV-4 and one patient with DENV-1 and DENV-3). A total of only 49 patients had successful virus isolation, 31had DENV-1, 10 had DENV-3 and 8 had DENV-4 based on RT-PCR results. DENV-2 was neither isolated nor detected by both RT-PCR and RT-qPCR.

Viremia levels were measured in 85 RT-qPCR positive patients and their viral load levels were compared. In comparing the viremia levels among these patients, the viremia levels of those three patients with co-infections were included in the analysis. There was no difference in the viremia levels of patients infected with any of the three DENV serotypes (Table 1). However, viremia levels among patients with DENV-3 (n = 11) infections were lower than among those with DENV-1 (n = 49) or DENV-4 (n = 28) infections, though there was no statistically significant difference among the three groups. Regardless of the infecting serotype(s), the viremia levels of patients with primary infection (n = 40) were higher than those with secondary infection (n = 45). If the infecting serotype was considered viremia levels of those patients with primary infection due to DENV-1 and DENV-3 but not due to DENV-4 also had higher viremia levels compared to those patients with secondary infections and having the same infecting serotype (Table 2). However, there was no statistically significant difference between these groups. Viral load levels were also compared among patients in the three disease severity categories, but no difference was found among the three groups (Table 3).

**Table 1:** Comparison of viremia levels among patients infected with different serotypes of DENV.

Dengue-1 (n = 49)	Dengue-3 (n = 11)	Dengue-4 (N = 28)	P value
Median (IQR)	Median (IQR)	Median (IQR)	
6.0x10^6^	8.0x10^5^	4.0x10^6^	0.21
(1.0x10^6,^-7.2x10^7^)	(4.0x10^5-^8.0x10^6^)	(9.2x106–7.5x10^7^)	

**Table 2:** Comparison of viremia levels of patients with primary and secondary DENV.

Patients	Primary Infection	Secondary Infection	P value
	Median (IQR)	Median (IQR)	
All DEN patients regardless of the infecting serotype^a^	4.0x10^6^	3.0x10^6^	0.48
	(7.0x10^5^-8.0x10^7^)	(3.710^5^-2x10^7^)	
DENV-1 infected patients^b^	1.5x10^7^	3.0x10^6^	0.22
	(1.0x10^6^-3.0x10^8^)	(1.0x10^6^,2.0x10^7)^	
DENV-3 infected patients^c^	2.4x10^6^	8.0x10^5^	0.88
	(5.7x10^5^,5x10^6^)	(4.0x10^5^-1.0x10^7^)	
DENV-4 infected patients^d^	3.0x10^6^	4.0x10^6^	0.76
	(1.0x10^5^-2.4x10^7^)	(9.7x10^4^-1.6x10^8^)	

Primary Infection was determined if Anti-DENV-IgG titers was below <29,000 titer

Secondary Infection was determined if Anti-DENV-IgG titers was equal and more than 29,000.

^a^ All DEN patients, primary infection = 40 patients and secondary infection = 45 patients (including co-infection cases)

^b^ DENV-1 infected patients, primary infection (n = 26), secondary infection (n = 23)

^c^ DENV-3 infected patients, primary infection (n = 6), secondary infection (n = 5)

^d^ DENV-4 infected patients, the primary infection (n = 11), secondary infection (n = 17)

**Table 3:** Comparison of viremia level according to severity of infection among all DENV patients.

Patients	DwoWS	DwWS	SD	P value
	Median (IQR)	Median (IQR)	Median (IQR)	
All DENV regardless of the infecting serotype^a^	9.0x10^6^	3.0x10^6^	4.0x10^6^	0.60
	(6.0x10^6^-2.0x10^7^)	(4.0x10^5^-5.0x10^7^)	(3.0x10^5^- 5.0x10^7^)	
DENV-1^b^	1.4x10^7^	6.0x10^6^	1.0x10^6^	0.90
	(4.5x10^6^-2.0x10^7^)	(2.0x10^6^-5.7x10^7^)	(6.5x10^5^-5.5x10^8^)	
DENV-3^c^	-*	7.5x10^5^	1.2x10^7^	0.63
		(4.0x10^5^-3.9x10^6^)	(8.0x10^6^, 1.6x10^7^)	
DENV-4^d^	1.8x10^7^	3.0x10^6^	2.5x10^6^	0.46
	(1.2x10^7^-2.4x10^7^)	(1.4x10^5^-6.0x10^8^)	(9.5x10^4^-5.2x10^6^)	

Dengue without warning signs- DwoWS;Dengue with warning signs-DwWS; Severe dengue-SD. *No case of DENV-3.

^a^ All DENV infected patients, DwoWS (n = 10), DwWS (n = 58) and SD (n = 17)

^b^ DENV-1infected patients, DwoWS (n = 7), DwWS (n = 31) and SD (n = 11)

^c^ DENV-3 infected patients, DwWS (n = 9) and SD (n = 2)

^d^ DENV-4 infected patients, DwoWS (n = 3), DwWS (n = 20) and SD (n = 5)

### Phylogenetic analysis

**Fig 1:**
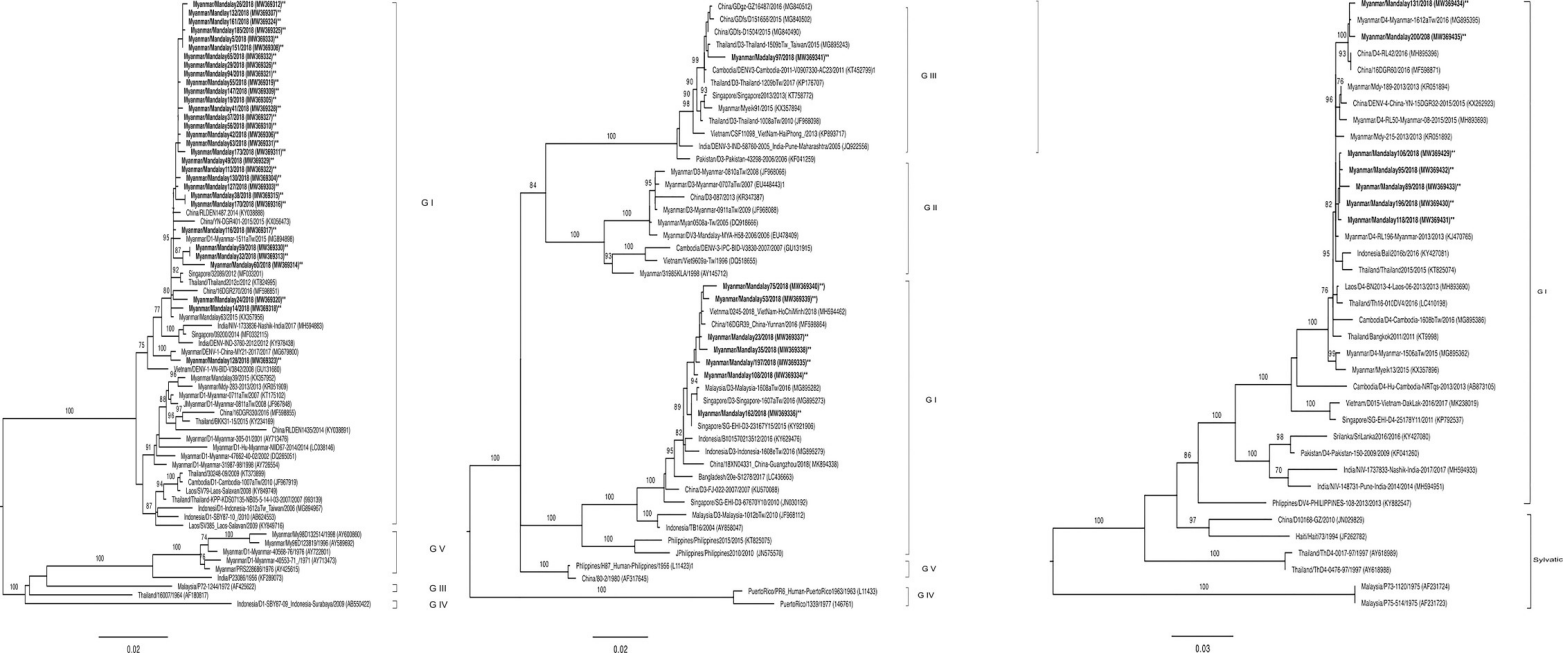
Phylogenetic trees based on the full coding region of the envelope protein gene of (A) DENV-1 (B) DENV-3 and (C) DENV-4. The representative strains of each genotype obtained from Genbank were named by country origin, strain name, year of isolation and GenBank accession number.

Phylogenetic trees analysis was performed based on the nucleotide sequences coding on the region of envelope protein gene. The nucleotide sequences were read by next-generation sequencing. DENV-1 belonged to two clades of genotype-1 and was similar to the strains circulating in China, Thailand, Singapore, and India and to the previously circulating strains in Myanmar (Fig 1A). For DENV-3, two genotypes (genotype-3 and the first time to be detected, genotype-1) were isolated in this study. Only one isolate of genotype-3 was seen, and the others belonged to genotype-1 which was the dominant genotype of DENV-3 in this study. For DENV genotype-3, this strain was closely similar to the viruses circulating in Thailand, China, Cambodia and to the strains detected in Myanmar in 2015 (Fig 1B). Genotype-1 was first detected in Mandalay and those strains were 99% similar to the strains circulating in China and several South East Asian countries (Vietnam, Singapore and Malaysia) For DENV-4, genotype-1 was circulating at the study area during this period. DENV-4 strains were also similar to the strains circulating in China, Indonesia, and Thailand and to native strains that had circulated in previous years (Fig 1C).

**Fig 2:**
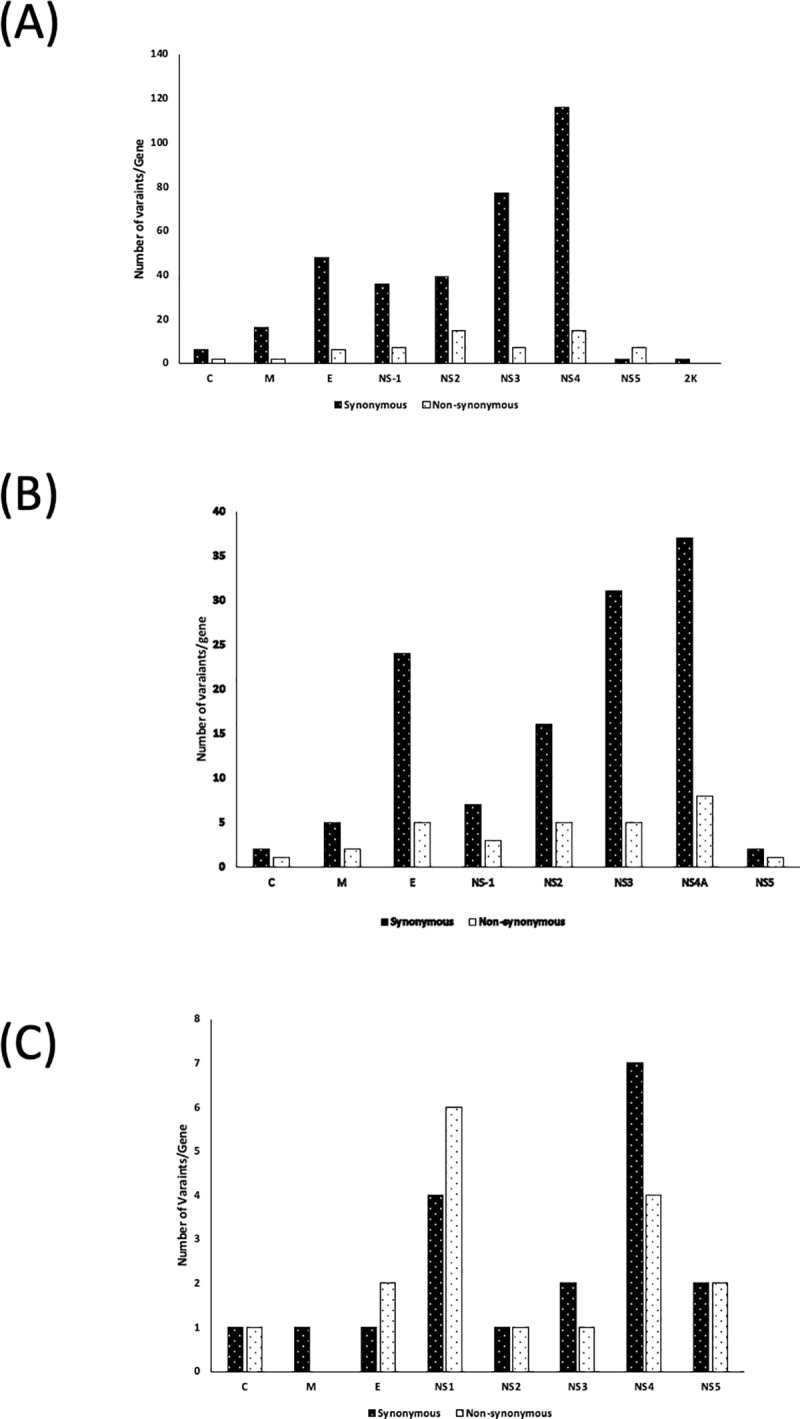
Number of positions with variant incidence > 1% per gene among the DENV-isolates. (A) DENV-1, (B) DENV-3 and (C) DENV-4.

Whole-genome sequencing was conducted on 16 viral strains for DENV-1, 7 strains for DENV-3 and 3 for DENV-4. The viral strains were chosen based on the disease severity of DEN patients and result of phylogenetic analysis. Amino acid variability analysis was performed on the whole genomes of selected viral isolates for all three serotypes of DENV isolated in this study. The DENV-1 strain from China (GenBank accession no. MG679800) was used as a reference strain in this study. For DENV 3, the viral strains isolated from China in 2017 (GenBank accession no. MN018387) and for DENV-4 the viral strain detected in China in 2015 (GenBank accession no. KY672957) were used as reference strains for analysis. A total of 593 mutations (485 synonymous and 108 non-synonymous mutations) were detected among all serotypes of DENV in this study. Synonymous mutation rates were higher than non-synonymous mutations among all genes of DENV serotypes (Fig 2). Moreover, both synonymous and non-synonymous mutations were higher in non-structural proteins than in structural proteins. Among the 16 DENV-1 isolates, a total of 403 mutations (342 synonymous and 61 non-synonymous mutations) were identified. Fifteen isolates of DENV-1 showed a stop codon mutation at amino acid position 3501(Y3501*) at NS5 region, and 14 of these isolates had a stop codon mutation at position 3483 (Q3483*) (Table 4). For DENV-3, a total of 154 mutations (124 synonymous and 30 non-synonymous mutations) were identified among 7 isolates. Furthermore, 36 mutations (19 synonymous and 17 non-synonymous) were identified among 3 isolates of DENV-4. The non-synonymous mutations among DENV-3 and DENV-4 isolates are shown in Tables 5 and 6, respectively.

**Table 4:** Non-synonymous variant (>1%) alleles shared among the DENV-1 isolates in this study.

Sample ID	Reference	Nucleotide Position	Amino acid position	Reference Allele	Alter Allele	Frequency	Change
Sample-5,6	C	182	30	G	A	99–100%	A-T
Sample-2,3,4	C	425	111	A	G	98–100%	T-A
Sample-13	M	612	173	C	T	100%	A-V
Sample-13	M	682	196	A	T	100%	Q-H
Sample-12	E	1125	344	A	G	100%	K-R
Sample-11	E	1400	436	A	T	58%	T-S
Sample-14	E	1742	531	T	C	1%	V-A
Sample-4	E	1811	573	A	G	80%	T-A
Sample-11	E	1895	601	C	A	100%	L-I
Sample-1,2,3,4,5,6,7,8,9,10,11,12,13,15,16	E	2079	662	T	C	96–100%	V-A
Sample-14	NS1	2656	836	T	A	100%	S-T
Sample-1,2,3,4,7,8,10,11,15,16	NS1	2711	873	G	A	96–100%	A-T
Sample-8	NS1	2712	873	C	T	100%	A-V
Sample-1,2,3,4,5,6,7,8,9,10,11,12,13,15,16	NS1	2715	874	G	A	97–100%	R-Q
Sample-1,2,3,5,6,7,8,9,10,11,12,13,15,16	NS1	3078	995	A	T	94–100%	D-V
Sample-13	NS1	3254	1054	T	G	100%	L-V
Sample-13	NS1	3288	1065	A	G	95%	H-R
Sample-1,2,3,4,5,6,7,8,9,10,11,12,13,15,16	NS2A	3815	1241	A	G	98–100%	M-V
Sample-1,2,3,4,5,6,7,8,9,10,11,12,13,15,16	NS2A	4043	1317	G	A	98–100%	A-T
Sample-1,2,3,4,5,6,7,8,9,10,11,12,13,15,16	NS2A	4085	1331	G	C	98–100%	V-L
Sample-1,3,5,7,8	NS2A	4112	1340	G	A	96–100%	G-R
Sample1,3,5,7,8,9,16	NS2A	4113	1340	G	A	80–100%	G-E
Sample-1,2,3,5,6,7,8,9,10,11,12,13,15,16	NS2B	4142	1350	G	A	98–100%	D-N
Sample-1,2,3,5,6,7,8,9,10,11,12,13,15,16	NS2B	4158	1355	G	C	94–100%	G-A
Sample-13	NS2B	4168	1358	A	G	100%	I-M
Sample-1,2,3,5,6,7,8,9,10,11,12,13,15,16	NS2B	4184	1364	G	A	97–100%	G-R
Sample-1,2,3,5,6,7,8,9,10,11,12,13,15,16	NS2B	4187	1365	G	T	98–100%	A-S
Sample-1,2,3,5,6,7,8,9,10,11,12,13,15,16	NS2B	4196	1368	G	A	66–95%	E-K
Sample-1,2,3,5,6,7,8,9,10,11,12,13,15,16	NS2B	4199	1369	G	A	98–100%	G-S
Sample-1,2,3,5,6,7,8,9,10,11,12,13,15,16	NS2B	4200	1369	G	A	98–100%	G-D
Sample-1,2,3,5,6,7,8,9,10,11,12,13,15,16	NS2B	4215	1374	G	C	98–100%	G-A
Sample-1,2,3,5,6,7,8,9,10,11,12,13,15,16	NS2B	4454	1454	G	A	96–100%	V-M
Sample-1,2,3,5,6,7,8,9,10,11,12,13,15,16	NS3	4748	1552	G	A	98–100%	V-L
Sample-12	NS3	4790	1566	A	G	100%	T-A
Sample-12	NS3	4944	1617	G	T	100%	R-I
Sample-14	NS3	5071	1641	G	T	0%	A-S
Sample-7,8,10,15	NS3	5127	1678	G	A	100%	R-K
Sample-9,12	NS3	5514	1807	T	C	100%	V-A
Sample-1,2,3,7,8,10,15,16	NS3	5830	1912	T	A	89–99%	D-E
Sample-1,2,3,5,6,7,8,9,10,11,12,13,15,16	NS4A	6380	2096	A	G	95–100%	I-V
Sample-13	NS4A	6644	2184	G	A	100%	A-T
Sample-1,2,3,4,5,6,7,8,9,10,11,12,13,15,16	NS4A	6654	2187	T	C	93–100%	V-A
Sample-13	NS4B	6885	2264	T	C	100%	V-A
Sample-9	NS4B	6906	2271	C	G	100%	A-G
Sample-7	NS4B	7256	2388	A	G	8%	K-E
Sample-7	NS4B	7917	2608	C	T	97%	P-L
Sample-12	NS4B	8080	2662	A	C	100%	E-D
Sample-3,4	NS4B	8706	2871	G	A	100%	R-K
Sample-2,3	NS4B	8718	2875	G	C	100%	S-T
Sample-16	NS4B	8880	2929	G	A	99%	R-K
Sample-1	NS4B	9236	3048	A	T	99%	I-F
Sample-1	NS4B	10001	3303	T	G	99%	S-A
Sample-5,6	NS4B	10173	3360	A	G	98%	N-S
Sample-1	NS4B	10209	3372	C	T	63%	S-L
Sample-5,6	NS5	10321	3409	A	C	100%	Q-H
Sample-1,2,3,4,5,6,7,8,9,10,11,12,13,15,16	NS5	10347	3418	T	C	96–100%	V-A
Sample-5,6	NS5	10352	3420	T	C	100%	Q-*
Sample-1	NS5	10446	3451	A	G	100%	H-R
Sample-1,2,3,4,6,7,8,9,10,11,12,13,15,16	NS5	10541	3483	C	T	99–100%	Q-*
Sample-1,2,3,4,5,6,7,8,9,10,11,12,13,15,16	NS5	10597	3501	T	G	100%	Y-*
Sample-1,2,3,4,5,6,7,8,9,10,11,12,13,15,16	NS5	10620	3509	C	T	98–100%	T-I

Sample-1 –Mdy-127/2018 Sample-2 –Mdy-132/2018 Sample 3- Mdy-151/2018

Sample-4 –Mdy-26/2018 Sample-5 –Mdy-38/2018 Sample 6- Mdy-170/2018

Sample-7 –Mdy-56/2018 Sample-8 –Mdy-147/2018 Sample 9- Mdy-32/2018

Sample-10 –Mdy-42/2018 Sample-11 –Mdy-130/2018 Sample 12- Mdy-60/2018

Sample-13 –Mdy-14/2018 Sample-14– Mdy-116/2018 Sample 15- Mdy-19/2018

Sample-16 –Mdy-173/2018

**Table 5:** Non-synonymous variant (>1%) alleles shared among the DENV-3 isolates in this study.

Sample ID	Reference	Nucleotide Position	Amino acid position	Reference Allele	Alter Allele	Frequency	Change
Sample-1,2	C	372	93	A	G	100%	N-S
Sample-3	M	470	126	A	G	100%	M-V
Sample-1	M	794	234	A	G	100%	I-V
Sample-4	E	1176	361	T	C	100%	I-T
Sample-1	E	1402	436	G	T	100%	Q-H
Sample-5	E	1749	552	G	A	100%	G-E
Sample-1,2,3,4,5,6	E	2018	642	T	C	100%	F-L
Sample-6	E	2073	660	T	C	100%	I-T
Sample-4	NS1	2805	904	C	T	95%	S-F
Sample-2	NS1	2936	948	T	C	100%	Y-H
Sample-2	NS1	3062	990	C	T	100%	L-F
Sample-7	NS2A	3636	1181	C	T	100%	T-I
Sample-1,2,3,4,5,6	NS2A	3673	1193	A	G	100%	I-M
Sample-5	NS2A	3867	1258	C	T	100%	A-V
Sample-1	NS2B	4334	1414	T	A	8%	S-T
Sample-4	NS2B	4353	1420	C	T	100%	T-I
Sample-2	NS3	5031	1646	A	G	100%	D-G
Sample-1	NS3	5561	1823	A	G	100%	N-D
Sample-1,2	NS3	5580	1829	A	C	100%	N-T
Sample-2	NS3	6012	1973	A	G	100%	N-S
Sample-3	NS3	6267	2058	A	G	100%	K-R
Sample-5	NS4A	6587	2165	A	G	100%	I-V
Sample-1	NS4B	7721	2543	C	T	100%	H-Y
Sample-3	NS4B	9204	3037	A	G	100%	H-R
Sample-1,2,3,4,5,6	NS4B	9229	3045	C	A	96–100%	H-O
Sample-4	NS4B	9408	3105	A	G	100%	E-G
Sample-3	NS4B	9512	3140	A	G	100%	K-E
Sample-1	NS4B	9599	3169	C	T	100%	L-F
Sample-1	NS4B	10019	3309	G	A	98%	E-K
Sample-4	NS5	10275	3394	A	G	100%	E-G

Sample-1 –Mdy-75/2018 Sample-2 –Mdy-108/2018 Sample 3- Mdy-23/2018

Sample-4 –Mdy-53/2018 Sample-5 –Mdy-35/2018 Sample 6- Mdy-197/2018

Sample-7 –Mdy-162/2018 Sample-8 –Mdy-147/2018 Sample 9- Mdy-32/2018

**Table 6:** Non-synonymous variant (>1%) alleles shared among the DENV-4 isolates in this study.

Sample ID	Reference	Nucleotide Position	Amino acid position	Reference Allele	Alter Allele	Frequency	Change
Sample-1,2,3	C	397	99	A	G	100%	K-R
Sample-1,2,3	E	996	299	G	T	98 = 100%	G-W
Sample-2	E	1209	370	A	G	100%	I-V
Sample-2	NS1	2455	785	A	G	100%	K-R
Sample-2	NS1	2878	926	T	C	94%	F-S
Sample-3	NS1	3093	998	C	A	100%	L-M
Sample-1,2,3	NS1	3293	1064	T	A	100%	D-E
Sample-1,2,3	NS1	3376	1092	T	C	100%	M-T
Sample-3	NS1	3388	1096	G	A	100%	R-K
Sample-1,2,3	NS2A	3748	1216	C	T	99–100%	T-I
Sample-1,2,3	NS3	5533	1811	A	G	97–100%	K-R
Sample-1,2,3	NS4B	8017	2639	Y	C	100%	X-S
Sample-2	NS4B	8178	2693	G	A	100%	V-I
Sample-1,2,3	NS4B	8407	2769	M	A	100%	X-Q
Sample-1	NS4B	9490	3130	G	A	92%	R-K
Sample-1,2,3	NS5	10309	3403	C	T	100%	P-L
Sample-2	NS5	10468	3456	G	A	10%	R-K

Sample-1 –Mdy-95/2018 Sample-2 –Mdy-106/2018 Sample 3- Mdy-118/2018

## Discussion

In this study, we conducted virological, serological and molecular characterization of DENVs that circulated in 2018 in Mandalay, Upper Myanmar. Our previous studies reported molecular epidemiology of DENVs in the same study area since 2006 [4–6]. This study revealed that 32% (48/148) and 7% (11/148) and 19% (28/148) of the patients examined were positive with DENV-1, DENV-3 and DENV-4 respectively.

The link between viremia and disease severity is still a controversial issue. Some studies showed an association between a high viremia level and disease severity, but this result was not universal [23–25]. Regarding the viremia level of patients, there was no difference among infecting serotypes, immune status, and severity levels in this study. The results were also similar to the findings of our previous study conducted in 2015 in Myanmar [4]. The viral load level among patients with primary infection was high and in some cases in patients with DEN encephalitis with primary infection had viral load levels that reached to 5.0 x 10^9^/mL; this result mirrored our findings in 2015 [4]. The viremia level was also consistently high up to 7 days post-infection in the 2015 study, and it could be the source of infection that caused outbreaks. In this study, there were nine cases (26.4%) of primary infection with the patients having SD. Previous studies also reported that proportion of SD patients with primary infection was high, perhaps it was because of the high viral load levels, genetic factors, and nutritional status of the patients [5].

One major DENV outbreak occurred during 2001 in Myanmar, and DENV-1 was replaced with the other three serotypes [26]. In 2006, only two cases of DENV-3, genotype-2, were identified [6] and DENV-3 then disappeared in Mandalay during 2015 DENV outbreak [4]. The number of DENV-3 infected cases increased in 2018 with the introduction of genotype-1 to Myanmar. Furthermore, DENV-1 has continued to circulate in Mandalay as the dominant serotype for more than one decade in Mandalay [4, 5]. In 2018, DENV-1 was circulating in the study area as dominant serotype. Cases of DENV-3 and DENV-4 infections were also increased compared to their prevalence in 2013 and 2015 whereas cases of DENV-2 infection were not detected [4].

The asymptomatic DENV infection rate is also important because asymptomatic person can transmit virus to mosquitoes. It will serve as source of infection through vectors [27]. One study conducted in Mandalay in 2018, the same period as that of the current study, identified six cases of asymptomatic DENV-1 infected children in Mandalay [28]. DENV-1 was also the dominant serotype among inapparent DENV infected patients. One study that conducted a complete genome analysis of DENV isolated during an outbreak in 2017 in Xishuangbanna, an autonomous prefecture in Southern China that borders Myanmar, Laos, also described a DENV-1 dominant outbreak that occurred in that region [29].

Three distinct clades of DENV-1 genotype-1 circulating in Myanmar was noted in 2013 and 2015 outbreaks. However, only two clades were noted in the 2018 DENV isolates in this study. In 2001 DENV outbreak in Myanmar, lineage extinction of genotype-1 of DENV-1 was noted during the 2001 DENV outbreak [30]. In a study conducted in Yunan Province (China) in the area bordering Myanmar and laos, three sub-clades of DENV-1 genotype-1 were also detected in 2013–2015 [31]. Among these three sub-clades, the viral strains that belonged to two sub-clades showed close similarities to the strain circulating in Myanmar in 2013 and 2015.

In this study, both DENV-3 genotype-1 and genotype-3 were detected, but genotype-1 was dominant, and its detection in Myanmar for the first time to mark this genotype’s first emergence in the country. One study in Yunan described the detection of both genotype-1 and genotype-3 of DENV-3 in travelers (both Myanmar and Chinese citizens) since 2017 [32]; a likely cause for this finding might have been the circulation of genotype-1 of DENV-3 in 2017, which was outside the scope of our study. Replacement of genotype-1 of DENV-3 from genotype-2 was also noted in Bangladesh [33]. Moreover, the greatest DENV outbreak occurred with DENV-2 (dominant genotype: cosmopolitan genotype and DENV-3 in Sri Lanka occurred in 2017. Genotype-1 of DENV-3 serotype was also first detected in Sri Lanka in 2017 [33–35]. In a surveillance study in Taiwan, on travelers from Southeast Asian countries between 2011 and 2016, genotype-1 of DENV-3 was seen from among travelers from Singapore, Malaysia, Indonesia, and the Philippines, but only genotype-2 and genotype-3 were reported in travelers from Myanmar and its neighbors (e.g., Laos, Cambodia, Bangladesh and India) [36]. Based on this surveillance, it is likely that genotype-1 of DENV-3 was circulating in Indonesia, the Philippines, Singapore and Malaysia before 2016 and that DENV-3 genotype-3 was predominant in Laos, India during that time [37, 38]. After 2017, genotype-1 was first introduced to Myanmar and neighboring countries. For DENV-4, only genotype-1 was isolated in this study and genotype shifting has not been observed since 2013.

Amino acids comparison was performed for all three DENV serotypes. Both synonymous and non-synonymous mutation rates among DENV-1 were higher than among the other two serotypes in this study. Among 16 isolates of DENV-1, stop codon mutations were found at NS-5 regions (Q3483* and Y3501*) of 15 isolates. Previously, stop codon mutations were also identified at envelope protein region but not at NS-5 regions of Myanmar DENV isolates detected in 2001 [39]. Those stop codon mutant strains were spread as defective RNA viruses and circulated among both human and mosquitoes. Although most defective RNA genomes were mostly found in cases of chronic virus infection, one study confirmed that defective RNA viruses are also responsible for acute DENV infection [40]. Moreover, defective RNA viruses play an important role in virus-host interaction because the defective variants can escape from immune response by means of adaptation, immune escape and virus perpetuation [41]. Further studies are required to confirm whether the stop codons containing DENV isolates were part of defective viral RNA. For DENV-3, only three non-synonymous mutations (F642L, I1193M and H3045O) were shared among six isolates. Among DENV-4, nine non-synonymous mutations were noted on all three isolates of genotype-1. Therefore, further in vitro and in vivo studies are needed to clarify the impact of the amino acid mutation in the viral genome related to clinical severity.

Myanmar is situated in South East Asia and borders five populous countries; Bangladesh, China, India, Laos, and Thailand. Routine surveillance of serotype but not genotypes, was conducted in Mandalay, the second biggest city in Myanmar and a major tourist attraction place close to the Chinese province Yunan. Populations from cross-border areas constituted a primary transmission source and are important for virus transmission and import of virus from neighboring countries. Globalization, urbanization, failure to control vectors and life style changes were the primary drivers behind DENV outbreaks [42]. As limitations, this study was conducted only among pediatric patients and only 26 virus isolates had whole genome sequencing.

## Conclusion

Three DENV serotypes were confirmed to be co-circulating during 2018 in Myanmar. Serotypes DENV-1 and DENV-4 persisted without genotype shift, and the number of DENV-3 cases increased with the first introduction of genotype-1. Pediatric patients with primary infection demonstrated high levels of viremia in this study. DENV-1 was dominant, and stop codon mutations were identified. The mutations rate was higher in non-structural regions than in structural ones. To our knowledge, this is the first report on complete genome analysis of DENV-3 and DENV-4 isolates among pediatric populations in Myanmar. Molecular surveillance is important, and annual surveillance is necessary to detect serotype and genotype shifts for early detection and prevention of outbreaks.
